# Effects of a Selective Cannabinoid CB2 Agonist and Antagonist on Intravenous Nicotine Self Administration and Reinstatement of Nicotine Seeking

**DOI:** 10.1371/journal.pone.0029900

**Published:** 2012-01-26

**Authors:** Islam Gamaleddin, Alexander Zvonok, Alexandros Makriyannis, Steven R. Goldberg, Bernard Le Foll

**Affiliations:** 1 Translational Addiction Research Laboratory, Centre for Addiction and Mental Health, Toronto, Canada; 2 Departments of Family and Community Medicine, Psychiatry, Pharmacology and Toxicology, Institute of Medical Science; University of Toronto, Toronto, Canada; 3 Addictions Program, Centre for Addiction and Mental Health, Toronto, Canada; 4 Department of Health and Human Services, Intramural Research Program, National Institute of Health, National Institute of Drug Abuse; Baltimore, Maryland, United States of America; 5 Center for Drug Discovery, Bouve College of Health Sciences, Northeastern University, Boston, United States of America; Baylor College of Medicine, United States of America

## Abstract

Over the last decade there have been significant advances in the discovery and understanding of the cannabinoid system along with the development of pharmacologic tools that modulate its function. Characterization of the crosstalk between nicotine addiction and the cannabinoid system may have significant implications on our understanding of the neurobiological mechanisms underlying nicotine dependence. Two types of cannabinoid receptors (CB1 and CB2) have been identified. CB1 receptors are expressed in the brain and modulate drug taking and drug seeking for various drugs of abuse, including nicotine. CB2 receptors have been recently identified in the brain and have been proposed to play a functional role in mental disorders and drug addiction. Our objective was to explore the role of CB2 receptors on intravenous nicotine self administration under two schedules of reinforcement (fixed and progressive ratio) and on nicotine seeking induced by nicotine priming or by nicotine associated cues. For this, we evaluated the effects of various doses of the selective CB2 antagonist AM630 (1.25 to 5 mg/kg) and CB2 agonist AM1241 (1 to 10 mg/kg) on these behavioral responses in rats. Different groups of male Long Evans rats were trained to lever press for nicotine at a unit dose of 30 µg/kg/infusion. Subsequently, animals were randomized using a Latin-square design and injected with either AM1241 or AM630 using a counterbalanced within subject design. Administration of the CB2 ligands did not affect either nicotine-taking nicotine-seeking behavior. Our results do not support the involvement of CB2 receptors in nicotine-taking or nicotine-seeking behavior.

## Introduction

Cigarette smoking is responsible for 5 million deaths worldwide every year. The mechanisms underlying tobacco smoking are of wide interest and clearly there is still a need for more effective medications to help in smoking cessation and prevent relapse [1]. The cannabinoid system appears to play a critical role in mediating the reinforcing effects of nicotine as well as relapse to nicotine-seeking behaviour. The cannabinoid system consists of CB1 and CB2 receptors, the endogenous cannabinoid receptor ligands, anandamide, and 2-arachidonoylglycerol (2-AG) [2], [3], in addition to the enzymes responsible for their degradation which are fatty acid amide hydrolase (FAAH) and monoacylglycerol lipase 2-AG, respectively [2], [4].

The CB1 receptor is highly expressed in the CNS, and is considered the most abundant G protein coupled receptor in the brain [5]. Cannabinoids act at CB1 receptors located presynaptically to elicit changes in the synaptic efficacy of central neuronal circuits that are involved in several processes including reward [6]. The CB2 receptors are predominantly expressed outside the central nervous system on immune tissues [7]. Recently, the expression of CB2 receptors has been reported in the brain. First, the expression of CB2 receptors was demonstrated in rat microglial cells and other cells in the brain associated with inflammation [8]–[11]. Then, CB2 receptor mRNAs were detected in rat brain (cerebellum, cortex, and brainstem) using reverse transcription polymerase chain reaction (RT-PCR) [12]. Moreover, CB2 receptor protein was detected using Western blotting and immunohistochemistry and evidence that CB2 receptors are functional and have antiemetic activity was obtained using intracranial ligand infusion [12]. More recently, it has been suggested that CB2 receptors may be involved in mental disorders and drug addiction [13], [14]. It has been reported that selective blockade of CB2 receptors prevented the development of alcohol preference, while selective activation of CB2 receptors enhanced alcohol preference, in mice subjected to chronic mild stress [13]. In addition, it has been recently reported that selective activation of CB2 receptors, reduced the reinforcing effects of cocaine and reduced levels of dopamine in the nucleus accumbens in wild-type and CB1 receptor knockout mice, but not in CB2 receptor knockout mice [15]. These findings support the notion that CB2 receptors are involved in modulating the reinforcing effects of drugs of abuse.

Most of the studies conducted so far, have explored the effects of activation or inactivation of CB1 receptors on drug-taking and drug-seeking behavior for various drugs of abuse, including nicotine. [16]–[21]. However, to our knowledge, no studies have examined the role of CB2 receptors on nicotine-taking and reinstatement of nicotine-seeking behavior. Here, we explored the impact of selective blockade and/or activation of CB2 receptors on nicotine self-administration behavior under fixed-ratio and progressive-ratio schedules of reinforcement and on reinstatement of nicotine-seeking behavior induced by reintroduction of nicotine-associated cues and by nicotine priming.

## Materials and Methods

### Animals

Male Long Evans rats (Charles River, Lachine, PQ, Canada) experimentally naive at the start of the study and initially weighing 250 to 275 g were used. All rats were individually housed in a temperature-controlled environment on a 12-h reverse light/dark cycle (lights off from 07:00 hours to 19:00 hours). Prior to any experimental manipulation, animals were given a minimum of 7 days to habituate to the colony room, during which they were weighed, handled and received unlimited access to both food and water. After habituation, all rats were diet restricted to 5 Pellets or 20 gms daily and had free access to water. Food restriction continued until all the experiments were completed. All the experimental procedures described in this report were carried out in compliance with the guidelines of the Canadian Council on Animal Care (compatible with NIH guidelines), and were reviewed and approved by the Centre for Addiction and Mental Health (CAMH) Animal Care Committee (Protocol no. 543).

### Apparatus

Nicotine intravenous self-administration studies were carried out in commercially available experimental chambers (Med Associates, St. Albans, Vt., USA) enclosed in sound attenuating boxes and equipped with two levers, a house light and 2 cue lights, one located above each lever. For half the animals, the left lever was the active lever and for the other half the right lever was the active lever. Session start was signaled by the illumination of the house-light and presentation of the levers. Pressing on the active lever resulted in the delivery of nicotine (30 µg/kg/infusion) when schedule requirements were met, accompanied by dimming of the house light and illumination of the cue light above the active lever. This continued for 60 seconds (time out period), during which further pressing on the active lever was recorded but had no programmed consequences. Pressing on the inactive lever was recorded, but had no programmed consequences throughout the session.

### Experimental Procedures

#### Food-maintained behavior

Techniques for initial acquisition of food-maintained behavior were similar to those already reported [22]–[24]. Animals learned to lever press for food reinforcement on a continuous reinforcement (CRF) schedule, in which each press on the active lever resulted in the delivery of a 45 mg food pellet. During the acquisition sessions, the house light was on and pressing the active lever resulted in the delivery of food with no illumination of the cue light above the levers. Daily 1-h acquisition sessions were conducted for 5 days. Once food-maintained behavior was acquired, intravenous catheters were surgically implanted.

#### Intravenous catheterization

Surgical procedures for implantation of chronic intravenous catheters were similar to those reported previously [22], [24]. Briefly, catheters were implanted into the jugular vein, exiting between the scapulae. Surgery was performed under anesthesia induced by xylazine (10 mg/kg, intraperitoneal (IP) and ketamine hydrochloride (90 mg/kg, IP). Incision sites were infiltrated with the subcutaneous (SC) local anesthetic marcaine (0.125%). Buprenorphine was given for post-operative analgesia (0.03 mg/kg, SC), and a single dose of penicillin (30,000 units, IM) was administered at the completion of surgical procedures. Animals were allowed to recover for a 1-week period before starting drug self-administration sessions.

#### Self-administration procedures

Acquisition of nicotine self-administration behavior was performed under a fixed-ratio (FR) schedule of reinforcement at a unit dose of 30 µg/kg/infusion of nicotine base. Session duration was 60 min. The start of each 60 min session was signaled by illumination of the house light. In the presence of the illuminated house light, completion of the schedule requirement on the active lever (i.e. 1 to 5 lever presses under FR1 to FR5) resulted in the delivery of a nicotine infusion. Each infusion was followed by a time out (TO) period of 60 seconds, during which the house light was dimmed, the cue light above the active lever illuminated, and lever press responses had no programmed consequences.

During the first five days of acquisition, response requirements were FR1 (i.e., each active lever press during the time-in period resulted in the delivery of a nicotine infusion), then FR2 for three days, then increased to reach a final value of FR5. Training was continued until the self-administration behavior was stable and the animals had a 15–20 day history of nicotine self-administration. Self-administration sessions were conducted mostly 5 days a week.

#### Testing under the FR5 schedule of reinforcement

Animals were considered to have acquired stable nicotine self-administration when they (1) pressed the active lever more than twice the number of times they pressed the inactive lever, (2) received a minimum of 10 infusions per 1-h session and (3) had less than 20% variation in the number of infusions earned per session over 2 consecutive sessions. Once stability was reached, the animals were given I.P. injections of vehicle 30 minutes before the start of the session, to habituate them to the injection procedure for an additional three days. Rats were randomized using a Latin-square design and were then tested with vehicle (0 mg/kg) and different doses of AM630 or AM1241 in a counter-balanced, within-subject design. Drugs were administered intraperitoneally 30 min before the session. Two separate groups of animals were used, one for testing the effects of the CB2 agonist AM1241 (N = 10) and the other for testing the CB2 antagonist AM630 (N = 12) on nicotine self-administration behavior under the fixed-ratio schedule, with drugs or vehicle administered 30 min before the session. Animals in each group were allowed at least two days of stable responding before they were retested with a different dose of either AM630 or AM1241.

#### Testing AM1241 under the PR schedule of reinforcement

A separate group of animals (N =  8) was trained to self-administer 30 µg/kg/infusion nicotine under the FR1 schedule for 5 days, then the FR2 schedule for 3 days and the FR5 schedule for another 2 days and then were directly switched to a progressive-ratio (PR) schedule where the response requirement during the session increased with each successive injection. The response requirement progression was based on the formula 5e_(0.25 inj number)-5_, with the first two values replaced by 5 and 10 (modified from Roberts *et al.*, 1993 [25]). Thus, the response requirements for successive injections were 5, 10, 17, 24, 32, 42, 56, 73, 95, 124, 161, 208, etc.. PR sessions lasted a maximum of 4 h. However, if the animal ceased to press the active lever for 30 minutes, the session automatically ended and the last ratio completed by the animal was defined as the break point. The animals were allowed 10 days of nicotine self-administration under the PR schedule and testing was performed only after stabilization of the responding on the active lever for at least 2 consecutive sessions before testing with AM1241 compound began. All animals reached their break points during the 4-h sessions within this 10-day training period and testing of vehicle (0 mg/kg) and AM1241 (1,3 and 10 mg/kg, IP, 30 min before the session) was then performed.

#### Testing AM630 under the PR schedule of reinforcement

The same group of animals that were tested with AM630 under the FR schedule of reinforcement ( N = 12) were switched to the PR schedule. After stabilization of behavior under the PR schedule for 2 successive sessions, animals were tested using vehicle (0 mg/kg) and the highest dose of AM630 (5 mg/kg) in a counterbalanced, within-subject design, in a similar fashion to that described with AM1241. Only 7 animals completed testing under the PR schedule; 5 animals were excluded due to catheter blockade.

#### Extinction

After acquisition of nicotine self-administration behavior, as described above, an extinction phase was conducted by withholding nicotine and its associated cues (house light remained on and cue lights remained off throughout the session). Responses on the active and inactive lever were recorded, but had no programmed consequences. An extinction criterion was established for each animal individually and was defined as total active lever responses during the session being less than 20 presses. This extinction criterion had to be maintained for 2 consecutive days before testing. All animals reached the extinction criterion within an average of 12 extinction sessions.

#### Effects of AM1241 on cue induced reinstatement of nicotine-seeking behavior

All tests were carried out in a counter-balanced within-subject design. After each test, extinction was re-established until extinction criteria were obtained for at least two consecutive days. Animals (N = 11) were pretreated 30 min before the session with vehicle (0 mg/kg) and 1, 3 and 10 mg/kg AM1241 in a counterbalanced order to measure the effects of AM1241 on cue-induced reinstatement of nicotine-seeking behavior. Cue induced reinstatement tests were conducted under conditions identical to that of self-administration, except that responses on the active lever (under a FR5 schedule) resulted in contingent presentation of the cues (light above the active lever on and house-light off for 60 s) without nicotine availability (no infusions). Responses on the inactive lever were recorded but had no programmed consequences. The testing sessions lasted for 60 minutes.

#### Effects of AM1241 on nicotine induced reinstatement of nicotine seeking

A new group of animals (N = 13) underwent a similar acquisition and extinction training procedure, as described above with cue-induced reinstatement. This group was tested for effects of vehicle (0 mg/kg) and AM1241 (1, 3 and 10 mg/kg IP 30 min before the session) on nicotine-induced reinstatement. Nicotine priming was performed as in [26], [27] by administering 0.15 mg/kg nicotine SC,10 min before the start of the test session.

#### Effects of AM630 on cue-induced and nicotine-induced reinstatement of nicotine-seeking behavior

Two separate groups of animals were tested for effects of AM630 on reinstatement of nicotine-seeking behavior induced by cues (N = 9) and by nicotine priming (N = 9). Animals were pretreated with vehicle (0 mg/kg) and AM630 (1.25, 2.5 and 5 mg/kg) IP 30 minutes before the start of the session. Cue-induced reinstatement tests were conducted under conditions identical to that of self-administration, except that responses on the active lever (on an FR5 schedule) resulted in contingent presentation of the cues (light above the active lever on and house-light off for 60 s) without nicotine availability (no infusions). Responses on the inactive lever were recorded but had no programmed consequences. Testing the effects of AM630 on nicotine-induced reinstatement was performed as in [26] by administering 0.15 mg/kg nicotine SC, 10 min before the start of test session, in the same manner and using the same methodology as described above with AM1241. All extinction and reinstatement sessions lasted for 60 minutes.

### Data Analysis

The number of active and inactive lever presses and the number of nicotine infusions were recorded and analyzed. To analyze the effects of AM1241 and AM630 on the number of nicotine infusions earned under the FR and the PR schedules of reinforcement, one way ANOVA analysis was performed. For reinstatement studies, one-way repeated measures analysis of variance (ANOVA) was used to assess the effects of AM1241 and AM630 on reinstatement induced by nicotine priming and by nicotine-associated cues. Student-t test was used to assess the effect of 5 mg/kg of AM630 pretreatment compared to vehicle pretreatment on nicotine self-administration behavior under the PR schedule of reinforcement.

### Drugs

(-)Nicotine hydrogen tartrate (Sigma-Aldrich, St Louis, Mo., USA) was dissolved in saline, the pH was adjusted to 7.0 (±0.2), and the solution was filtered through a 0.22 mm syringe filter (Fisher Scientific, Pittsburgh, Pa., USA) for sterilization purposes. All nicotine doses are reported as free base concentrations. Nicotine was administered IV in a volume of 100 µl/kg/injection for self-administration studies or was administered SC at the dose of 0.15 mg/kg for reinstatement studies. AM1241 (2-iodo-5-nitrophenyl)-(1-(1-methylpiperdin-2-ylmethyl)-1 h-indol-3-yl) methanone was dissolved in 20% DMSO in saline and injected IP 30 min before the start of the session and was synthesized by the group of Dr. Alexandros Makriyannis, the Centre for Drug discovery at Northeastern University, Boston, MA, USA .AM630 (6-Iodo-2-methyl-1-[2-(4-morpholinyl)ethyl]-1*H*-indol-3-yl](4-methoxyphenyl)methanone) (Tocris Bioscience, Missouri USA) was dissolved in 10%DMSO, 10% tween in distilled water and injected IP in a volume of 1 ml/kg 30 min before the start of the session.

## Results

### Acquisition of nicotine self-administration behavior under fixed ratio schedule of reinforcement

**Figure 1:**
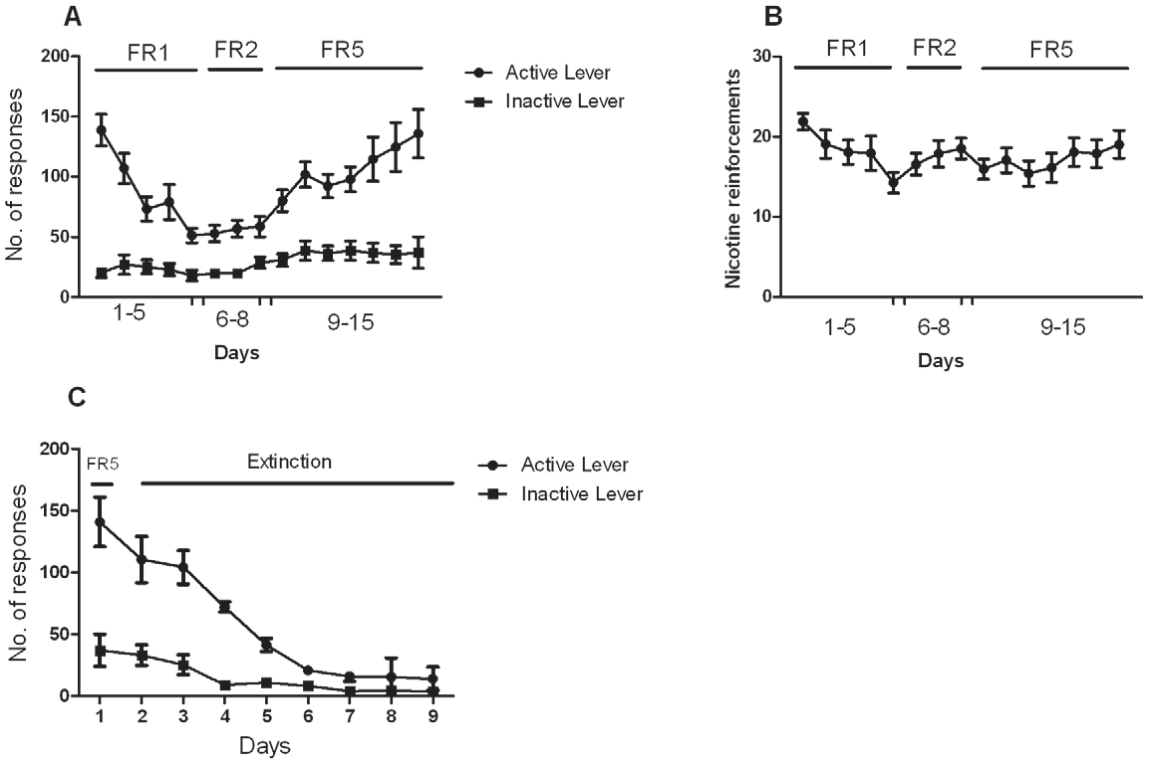
Pattern of respondinng during acquisition and extinction phases. A. Acquisition of nicotine self-administration (30 µg/kg/infusion). The total number of active (•) and inactive(▪) lever presses (means ± SEM) received in each session (during time in and time out periods) under the different schedules of reinforcement (FR- 1, FR-2, FR-5,). B. Number of nicotine infusions (means ± SEM) earned during acquisition phase in the same group of animals represented as fig. 1A. C. The number of active (•) and inactive(▪) lever presses (means ± SEM) received in each extinction session in the same group of animals represented in figures 1A & 1B.

During the first week of acquisition, responding on the active lever decreased to low levels, then gradually increased when the ratio requirement was increased up to FR5; in contrast, responding on the inactive lever remained low (Fig. 1A). Over the next 2 weeks, responding on the active lever under the FR5 schedule that was reinforced by nicotine infusion increased to the high levels previously maintained by food, while responding on the inactive lever remained low. The number of nicotine infusions throughout the different schedules of reinforcement (FR1 – FR5) showed a consistent level of nicotine self administration (above 10 infusions/session) (Fig. 1B).

### Extinction

The data presented in Fig. 1C reflect the extinction pattern for the group of animals (*N* = 12) used in the experiment testing the effect of AM1241 on cue-induced reinstatement (only 11 animals completed testing on cue-induced reinstatement and 1 animal was excluded due to failure of extinction). Most animals reached extinction criteria within 8–9 days and testing with AM1241 on reinstatement was started (extinction training was pursued for the remaining rats until they reached the extinction criteria).

### Effects of AM1241 on nicotine self-administration behavior under the FR5 schedule

**Figure 2:**
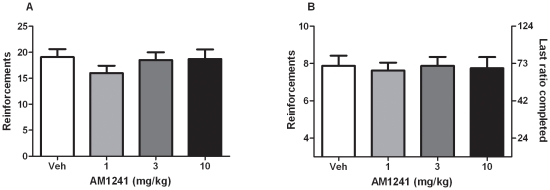
Effects of AM1241 on nicotine self-administration under FR5 and PR schedules of reinforcement. **A**. Effects of pretreatment with AM1241 (1, 3 and 10 mg/kg, IP H 30) on nicotine (30 µg/kg/infusion) self-administration under the FR5 schedule. Data are expressed as means (±SEM) of the number of nicotine infusions obtained during the 60-min session. All doses of AM1241 did not affect responding vs. vehicle (0 mg/kg) pretreatment (N = 10); P = 0.35. **B**. Effects of pretreatment with AM1241 (1, 3 and 10 mg/kg) on nicotine (30 ug/kg/infusion) self- administration under PR schedule. **A**, Data are expressed as means (±SEM) of the number of nicotine infusions obtained during the 4-hr sessions. AM1241 did not affect break point P>0.05 compared to vehicle (0 mg/kg) pretreatment. (N = 8) P = 0.89.

ANOVA analysis showed no significant effect of AM1241 pretreatment on the number of nicotine infusions (F_3, 27_ = 1.13, *P* = 0.35), and pair wise comparisons with vehicle (0 mg/kg) indicated that administration of AM1241 (1, 3 and 10 mg/kg) did not affect the number of nicotine infusions received during the session (N = 10) (Fig. 2A).

### Effects of AM1241 on nicotine self-administration behavior under the PR schedule

ANOVA analysis showed no significant effect of AM1241 pretreatment on the number of nicotine infusions (F_3, 21_ = 0.20, *P* = 0.89). Administration of various doses of AM1241 (1, 3 and 10 mg/kg) failed to produce any change in break point values, as compared to vehicle (0 mg/kg; N = 8) (Fig. 2B).

### Effect of AM1241 on reinstatement of nicotine-seeking behavior induced by nicotine-associated cues

**Figure 3:**
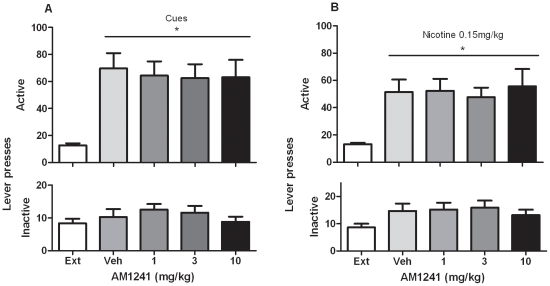
Effects of AM1241 on reinstatement of nicotine-seeking behavior induced by presentation of nicotine associated cues and by Nicotine priming. **A**. A significant reinstatement of nicotine-seeking behavior was produced by presentation of nicotine associated cues alone compared to extinction condition (Ext) (* P<0.001). ANOVA showed that pretreatment with AM1241 (1, 3 and 10 mg/kg, IP, H 30 min) did not modify cue induced reinstatement of nicotine-seeking behavior compared to vehicle (0 mg/kg) (P>0.05) N = 11. Data are expressed as means (±SEM) of the number of active and inactive lever presses during extinction (Ext); vehicle (0 mg/kg) pre-treatment (visual cues) and after pretreatment with AM121 (1, 3 and 10 mg). **B**. A significant reinstatement of nicotine-seeking was produced by pretreatment with nicotine (0.15 mg/kg) compared to extinction condition (Ext) (* P<0.001). ANOVA showed that AM1241 (1, 3, 10 mg/kg, IP, H 30 min) did not modify reinstatement of nicotine-seeking behavior induced by a priming injection of 0.15 mg/kg nicotine administered 1 min before the session compared to vehicle (0 mg/kg) pretreatment (P>0.05). Data are expressed as means (±SEM) of the number of active and inactive lever presses during extinction (Ext); vehicle (0 mg/kg) pre-treatment and after pretreatment with AM121 (1, 3 and 10 mg). N = 13.

ANOVA analysis performed on active lever presses indicated a main effect of cues per se on reinstatement of nicotine seeking compared to extinction (Ext) conditions (*P*<0.001). Newman-Keuls Multiple Comparison Test performed on the active lever presses indicated no effect of AM1241 administration (F_4, 40_ = 19.75; *P*>0.05), compared to cue-induced reinstatement after vehicle (0 mg/kg) administration. Neither presentation of nicotine-associated cues nor AM1241 administration, had a significant effect on responding on the inactive lever (F_4, 40_ = 1.34, *P* = 0.27) (N = 11)(Fig. 3A).

### Effect of AM1241 on reinstatement of nicotine-seeking behavior induced by nicotine priming

ANOVA analysis performed on active lever presses indicated a main effect of 0.15 mg/kg nicotine priming on nicotine-seeking behavior, as compared to extinction (Ext) conditions (*P*<0.001). ANOVA analysis performed on the active lever presses indicated no effect of AM1241 (F_3, 36_ = 6.64; *P*>0.05), as compared to nicotine-induced reinstatement after vehicle (0 mg/kg) pretreatment. Neither priming injections of nicotine, nor AM1241 (1, 3 and 10 mg/kg) administration, had a significant effect on responding on the inactive lever (F_3, 36_ = 1.80; *P* = 0.14) (N = 13)(Fig. 3B).

### Effects of AM630 on nicotine self-administration behavior under the FR5 schedule

**Figure 4:**
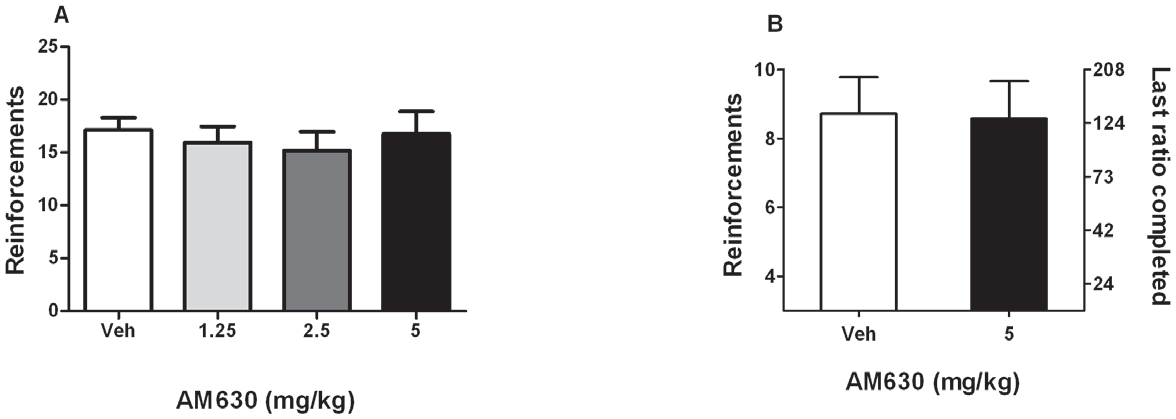
Effect of AM630 on nicotine self administration under FR5 and PR schedules of reinforcement. **A**. Effects of pretreatment with AM630 (1.25, 2.5 and 5 mg/kg, IP, H 30) on nicotine (30 µg/kg/infusion) self administration under the FR5 schedule. Data are expressed as means (±SEM) of the number of nicotine infusions obtained during the 60-min session. AM630 did not affect responding vs. vehicle (0 mg/kg) pretreatment (N = 12); P = 0.67. **B**. Effects of pretreatment with AM630 (5 mg/kg, IP) on nicotine (30 ug/kg/infusion) self administration under PR schedule. **A**, Data are expressed as means (±SEM) of the number of nicotine infusions obtained during the 4-hr sessions. AM630 did not affect break point P>0.05 vs. vehicle (0 mg/kg) pretreatment. (N = 7) P = 0.73.

ANOVA showed no effect of AM630 pretreatment on the number of nicotine infusions received during the session (F_3, 33_ = 0.51, *P* = 0.67), and pair wise comparisons with vehicle (0 mg/kg) pretreatment indicated that administration of AM630 (1.25, 2.5 and 5 mg/kg) did not affect the number of nicotine infusions received during the session (N = 12) (Fig. 4A).

### Effects of AM630 on nicotine self-administration behavior under the PR schedule

Student-t test showed no effect of AM630 pretreatment on the number of nicotine infusions received during the session (*P* = 0.73). Administration of 5 mg/kg AM630 failed to produce any change in the break point values, as compared to vehicle (0 mg/kg) (Fig. 4B)(N = 7).

### Effects of AM630 on reinstatement of nicotine-seeking behavior induced by nicotine-associated cues

**Figure 5:**
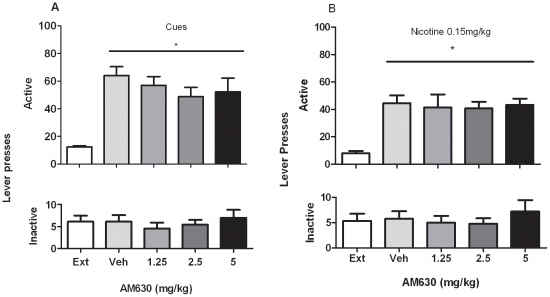
Effects of AM630 on reinstatement of nicotine-seeking behavior induced by presentation of nicotine associated cues and by Nicotine priming. **A**. Effects of pretreatment with AM630 (1.25, 2.5 and 5 mg/kg, IP H 30 min) on cue-induced reinstatement of nicotine-seeking behavior. A significant reinstatement of nicotine-seeking behavior was produced by presentation of nicotine-associated cues alone (* P<0.001). ANOVA showed that pretreatment with AM630 (1.25, 2.5 and 5 mg/kg, IP, H 30 min) did not modify cue induced reinstatement of nicotine-seeking behavior compared to vehicle (0 mg/kg) pretreatment (P>0.05). Data are expressed as means (±SEM) of the number of active and inactive lever presses during extinction (Ext); vehicle (0 mg/kg) pre-treatment and after pretreatment with AM630 (1.25, 2.5 and 5 mg). **B**. A significant reinstatement of nicotine-seeking was also produced by pretreatment with nicotine (0.15 mg/kg) (* P<0.001). ANOVA showed that AM630 (1.25, 2.5, 5 mg/kg, IP, H 30 min) did not modify reinstatement of nicotine-seeking behavior induced by a priming injection of 0.15 mg/kg nicotine administered 1 min before the session (P>0.05). Data are expressed as means (±SEM) of the number of active and inactive lever presses during extinction (Ext); vehicle (0 mg/kg) pre-treatment and after pretreatment with AM630 (1.25, 2.5 and 5 mg).

ANOVA analysis performed on active lever presses indicated a main effect of cues per se on reinstatement of nicotine-seeking behavior compared to extinction (Ext) conditions (*P*<0.001). ANOVA performed on the active lever presses indicated no effect on cue-induced reinstatement. of different doses of AM630 (1.25, 2.5 and 5 mg/kg) (F_4, 32_ = 14.94; *P*>0.05), compared to vehicle (0 mg/kg). Neither presentation of nicotine-associated cues, nor administration of AM630, had a significant effect on responding on the inactive lever (F_4, 32_ = 0.50 *P* = 0.73)( N = 9) (Fig. 5A).

### Effects of AM630 on reinstatement of nicotine-seeking behavior induced by nicotine priming

ANOVA analysis performed on active lever presses indicated a main effect of 0.15 mg/kg nicotine priming on nicotine-seeking behavior, compared to extinction (Ext) conditions (*P*<0.001). ANOVA analysis performed on the active lever presses showed no effects of administration of AM630 (1.25, 2.5 and 5 mg/kg) (F_4, 32_ = 8.33; *P*>0.05), as compared to vehicle (0 mg/kg) pretreatment. Neither priming injections of nicotine nor AM630 administration, had a significant effect on responding on the inactive lever (F_4, 32_ = 0.73; *P* = 0.57)(N = 9) (Fig. 5B).

## Discussion

This study is the first to evaluate the impact of selective CB2 receptor ligands on an animal model of nicotine-taking and nicotine-seeking behavior. Neither activation of CB2 receptors by the selective CB2 agonist AM1241, nor blockade using the selective CB2 antagonist AM630 produced significant effects on nicotine-taking behavior under fixed-ratio or progressive-ratio schedules of reinforcement. Moreover, both compounds failed to modulate nicotine-seeking behavior induced by reintroduction of nicotine-associated cues or by priming injections of nicotine just before the start of the session.

To our knowledge, data on the behavioral properties of AM1241 are relatively scarce and are mostly limited to studying its effects on motor function and pain. We selected a dose range that covers the different doses used in several previous studies [28]–[30], doses that had potent antinociceptive effects, but, no locomotor, cataleptic or motor side effects [31], [32]. Similarly, AM630 has seldom been tested in drug dependence paradigms. Similar to AM1241, we used a relatively wide range of AM630 doses similar to doses previously tested [33], [34]. Choice of AM630 was due to its high potency and affinity for rat CB2 receptors [35].

Our results with AM1241 on nicotine self administration under the fixed-ratio schedule of reinforcement are in agreement with previous results with the CB2 agonist JWH015, which failed to modulate alcohol intake in C57Bl/6 mice under a fixed-ratio schedule of reinforcement [13]. Furthermore, selective blockade of CB2 receptors by AM630 did not affect alcohol intake in the same strain of mice under the same schedule of reinforcement [13]. However, both JWH015 and AM630 were able to increase and decrease alcohol intake, respectively, in mice subjected to chronic mild stress, which is a paradigm outside the scope of this study [13]. These findings were later replicated by the same group which also reported that blockade of CB2 receptors decreased food consumption in C57Bl/6 mice but failed to produce significant changes in food intake for Balb/c and DBA/2 mice [36].

In contrast to our findings, Xi *et al.* have recently shown that systemic, intranasal and local intra-accumbens administration of the selective CB2 agonist JWH133, produced a dose dependent decrease in intravenous cocaine self-administration behavior, in cocaine-induced hyperlocomotion, and in cocaine-induced increases in extracellular levels of dopamine in the nucleus accumbens in wild-type and CB1 receptor knockout mice, but not in CB2 knockout mice [15]. The effects observed with CB2 receptor activation were reversed by the selective CB2 antagonist AM630 [15]. The difference between our findings and the findings by Xi and colleagues may be due to differences in the neurobiological substrates of the drug of abuse studied (nicotine vs. cocaine), differences in the role of CB2 receptors based on the animal strain (rats vs. mice), differences in the pharmacological effects of the CB2 agonist used (JWH133 vs. AM1241, or differences in the schedule of reinforcement used (FR5 vs. FR1). Further work addressing those factors would be needed to clarify the role of CB2 receptors in drug reinforcement.

The behavioral findings in this study are in apparent contrast with our recent findings that stimulation of CB1/2 receptors using the mixed CB1/2 receptor agonist WIN 55,212-2 increases nicotine self-administration behavior under a progressive-ratio schedule of reinforcement.

Moreover, in the same study, we demonstrated that administration of WIN 55,212-2 per se reinstates nicotine-seeking behavior, an effect that was reversed by the selective CB1 inverse agonist/antagonist rimonabant but not by the selective CB2 antagonist AM630, indicating that this enhancement of nicotine-seeking behavior was mediated by CB1 receptors. WIN 55,212 also significantly enhanced reinstatement of nicotine-seeking behavior induced by reintroduction nicotine associated cues, an effect that was also reversed by rimonabant [21].

The results in this study, along with our previous work on CB1 receptor stimulation, add more evidence to the current literature that CB1 and CB2 receptors have several distinct behavioral, neurochemical and immunological profiles, yet they overlap in some properties like antinociception, catalepsy (when higher doses are tested) [30], [37].

One limitation in this study is the lack of data on stress-induced reinstatement. This aspect would be worth exploring in further studies. It is clear that neurotransmitters, such as noradrenaline and corticotrphin releasing factor, are involved in mediating stress-induced reinstatement [38] and we cannot exclude an involvement of CB2 receptors in stress-induced reinstatement of nicotine-seeking behavior at this point.

In conclusion, the findings in this study provide evidence that CB2 receptors are not involved in the reinforcing effects of nicotine and in reinstatement of nicotine-seeking behavior induced by cues and nicotine priming in rats. In this study we used the intravenous mediated paradigm which has been previously used by us and several other laboratories to assess the pivotal role CB1 receptors play in the reinforcing effects of nicotine, yet in this study using the same paradigm we were not able to demonstrate a similar role of CB2 receptors on nicotine self-administration behavior or reinstatement of nicotine seeking behavior. Hence, we believe that ligands modulating the CB1 receptors (either directly or indirectly by modulating endocannabinoid tone) could potentially be a more useful tool than CB2 ligands in modulating the reinforcing and relapse related effects of nicotine [23], [39]–[41]. The findings in this study could be specific to nicotine and not generalizable to other drugs of abuse. Therefore, further studies are warranted to investigate the role of CB2 receptors on the reinforcing and relapse related effects different drugs of abuse.
